# Hydrogen-rich saline protects against ultraviolet B radiation injury in rats

**DOI:** 10.7555/JBR.26.20110037

**Published:** 2012-04-24

**Authors:** Ze Guo, Bingrong Zhou, Wei Li, Xuejun Sun, Dan Luo

**Affiliations:** a Department of Dermatology, the First Affiliated Hospital of Nanjing Medical University, Nanjing, Jiangsu 210029, China;; b Department of Diving Medicine, Faculty of Naval Medicine, Second Military Medical University, Shanghai 200433, China.

**Keywords:** ultraviolet B, photo-damage, oxidative stress, hydrogen-rich saline, skin

## Abstract

Exposure of skin to solar ultraviolet (UV) radiation induces photo-damage. Ultraviolet B (UVB) is the major component of UV radiation which induces the production of reactive oxygen species (ROS) and plays an important role in photo-damage. Hydrogen gas reduces ROS and alleviates inflammation. In this study, we sought to demonstrate that hydrogen-rich saline has the effect on skin injuries caused by UVB radiation. UVB radiation was irradiated on female C57BL/6 rats to induce skin injury. Hydrogen-rich saline and nitrogen-rich saline were administered to rats by intraperitoneal injection. Skin damage was detected by microscope after injury. UVB radiation had a significant affection in tumor necrosis factor alpha, interleukin (IL)-1β and IL-6 levels, tissue superoxide dismutase, malondialdehyde and nitric oxide activity. Hydrogen-rich saline had a protective effect by altering the levels of these markers and relieved morphological skin injury. Hydrogen-rich saline protected against UVB radiation injury, possibly by reducing inflammation and oxidative stress.

## INTRODUCTION

Although solar radiation is essential to human life, solar ultraviolet (UV) radiation, principally in the middle wavelength range (290-320 nm, UVB)[2], can cause skin photo-damage[1]. Over the last three decades, it has become increasingly clear that UVB irradiation induces the production of reactive oxygen species (ROSs)[3]. Excited electrons in oxygen interact with numerous molecules in the cell that may impact on many important biochemical processes[4]–[5]. These interactions lead to altered cell growth and differentiation. When generated in excess, ROS can also induce tissue injury and contribute to the development of skin cancer[6],[7]. Hydrogen (H_2_) is a potent free radical scavenger, which selectively reduces the hydroxyl radical, the most cytotoxic of ROS, and protects against tissue damages such as transient cerebral ischemia, neonatal cerebral hypoxia-ischemia, liver injury and ischemia-reperfusion injury of the heart, lung, intestine and kidney[8]–[14]. The therapeutic antioxidant activity of H_2_ depends mainly on its antioxidant properties. The use of H_2_ gas in clinical practice is advantageous due to its low cost compared with other anti-oxidative agents. However, H_2_ gas inhalation is difficult to apply because of its flammability and explosion hazards. By contrast, H_2_ gas-saturated norml saline, which is called hydrogen-rich saline, is easy to apply and safe. By examining the inflammation response and oxidative stress in rats, we investigated whether the administration of H_2_-rich saline exerted a protective effect in a animal model of UVB radiation injury in the current study.

## Materials and methods

### Animals

Adult female C57BL/6 rats, weighing from 220 to 250 g, were obtained from the Experimental Animal House of Nanjing Medical University (Nanjing, China) and housed in environmentally controlled conditions (22°C, a 12 h light/dark cycle with the light cycle from 6:00 to 18:00 and the dark cycle from 18:00 to 6:00) with ad libitum access to standard laboratory chow and water. Before their exposure to UVB irradiation, rats were allowed 48 h to adapt to their new environment. The study protocol was approved by the local institutional review board in the First Affiliated Hospital of Nanjing Medical University. Animal welfare and experimental procedures were carried out strictly in accordance with the Guide for Care and Use of Laboratory Animals (National Research Council of USA, 1996). A depilatory was used to remove the pelage from the back of the rats.

### Production of hydrogen-rich saline

H_2_-rich saline and nitrogen-rich saline were prepared by dissolving H_2_ and nitrogen in normal saline under high pressure (0.4 MPa) for 6 h to obtain supersaturated saline. The saturated H_2_ saline was stored under atmospheric pressure at 4°C in an aluminum bag with no dead volume. We used gamma irradiation to sterilize both H_2_-rich saline and nitrogen-rich saline. H_2_-rich saline was prepared freshly every week to ensure that the concentration was maintained at more than 0.6 mmol/L. Gas chromatography was used to confirm the content of hydrogen in saline as described by Ohsawa *et al*[9].

### Animal grouping and UVB irradiation

The rats were divided into four groups with 8 rats per group. In the control group, rats received normal feed only; in the UVB irradiation group, rats received UVB irradiation at 180 mJ/cm^2^ delivered an irradiation device (Sigma-Aldrich, St. Louis, MO, USA) which used a Philips TL/12 UVB light tube. In the H_2_-rich saline group, rats were injected with H_2_ gas-saturated normal saline at 5 mL/kg 10 min before and 8 h after irradiation. In the nitrogen-rich saline group, rats received an intraperitoneal injection of nitrogen-rich saline at 5 mL/kg 10 min before and 8 h after UVB irradiation. An UVB digital radiometer (Sigma-Aldrich) was used to confirm the dosage of UVB irradiation. The dose of UVB irradiation was based on our preiovus studies[15]–[17].

### Light microscopy

After rats were sacrificed, dorsal skin tissues were removed and fixed in 10% formaldehyde, and embedded in paraffin and stained with hematoxylin and eosin and examined under an Olympus BX50 microscope (Olympus, Tokyo, Japan).

### Measurement of superoxide dismutase and malondialdehyde

Rat dorsal skin tissues were stored immediately at -80°C and were homogenized before experiments. The homogenate was centrifuged at 1,084 *g* for 10 min, and the protein content in the supernatant was determined by Coomassie brilliant blue staining. Superoxide dismutase (SOD) activity was determined using a commercially avaialbe kit (Nanjing Jiancheng Bioengineering Institute, Nanjing, China) according to the manufacturer's instructions and expressed as vital units (U) in the skin tissue, which were defined as the amount of SOD per 1 mg tissue protein in 1 mL reaction solution when the SOD inhibition ratio was at 50%. The quantitative malondialdehyde (MDA) assay was performed as described by Ohkawa *et al*. [18], and MDA was measured spectrophotometrically at 532 nm.

### Measurement of nitric oxide

The skin tissue was homogenized as described above for SOD. Nitric oxide (NO) was spectrophotometrically assayed by measuring the accumulation of its stable degradation products, nitrate and nitrite. For accurate assessment of the total NO generated, nitrate was converted into nitrite by nitrate reductase, followed by the quantitation of nitrite using Griess reagent. NO was estimated by means of the total nitrite formed using a colorimetric NO assay kit (Nanjing Jiancheng Bioengineering Institute, Nanjing, China).

### Cytokine determination

Blood samples were drawn from the heart of the rats and centrifuged at 1,084 *g* for 15 min to obtain serum. Cytokine levels in the serum were determined using highly sensitive enzyme-linked immunosorbent assay kits (R&D Systems, Minneapolis, MN, USA) according to the manufacturer's recommendations.

### Statistical analysis

Data were expressed as mean±SD for each group. We used the SPSS for Windows Version 16.0 (SPSS Inc., Chicago, IL, USA) to analyze the data. A *P* value of less than 0.05 was considered statistically significant.

## RESULTS

### H_2_-rich saline treatment attenuates neutrophil infiltration

**Fig. 1:**
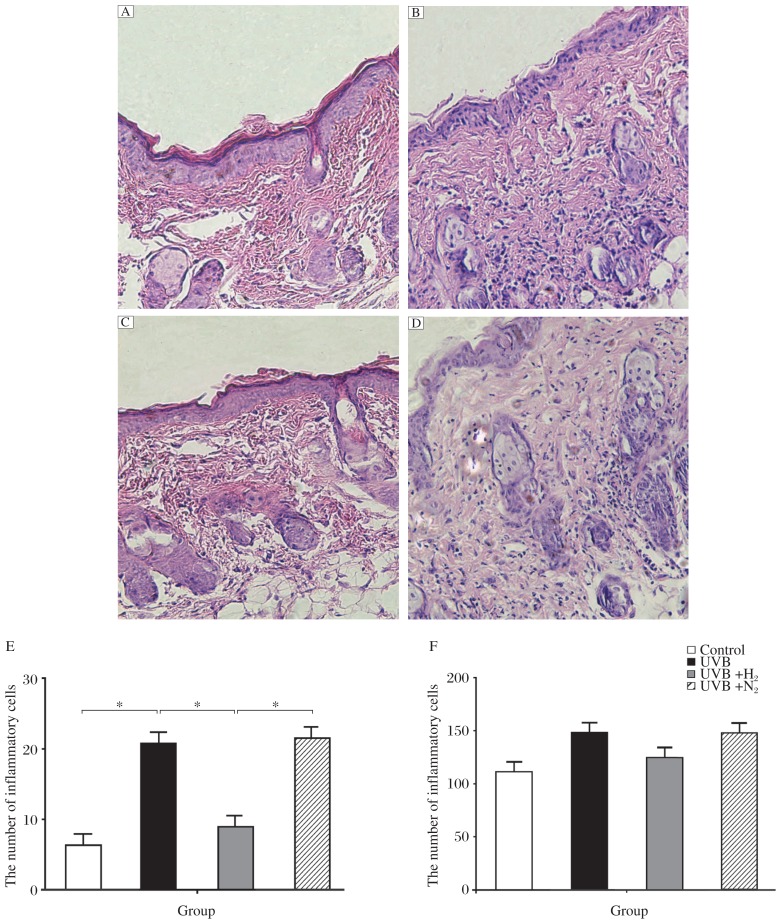
Morphologic observations of rat skin treated by UVB combined with H_2_-rich saline or nitrogen-rich saline. The skin tissue was stained with H&E (×200). A: the control group received no treatment. B: rats subjected to UVB irradiation. C: Rats were irradiated by UVB and injected with H_2_-rich saline. D: Rats were irradiated by UVB and injected with nitrogen-rich saline. The number of inflammatory cells in group A to D is shown in E. Skin thickness in group A to D is shown in F. Data are expressed as mean±SD, *n* = 8 per group. *P < 0.05.

We used hematoxylin and eosin (H&E) staining to observe the degree of inflammatory reaction in the skin tissue. Neutrophil infiltration is the typical characteristic of an acute inflammatory reaction. After UVB irradiation, massive neutrophil infiltration was found in the skin tissue, principally in the hypodermis (***Fig. 1A*** and ****1B****). However, after intraperitoneal injection of hydrogen-rich saline, neutrophil infiltration was not as great (***Fig. 1A*** and ****1B****). Administration of nitrogen-rich saline did not reduce neutrophil infiltration (***Fig. 1A*** and ****1D****). We used light microscope to observe the degree of inflammation in mouse skin tissue, and three visual fields were chosen from every section to calculate the number of inflammatory cells and a mean value was obtained (***Fig. 1B***). There was a statistical difference between group b and group c, and no statistical difference was noted in skin thickness between the four groups (***Fig. 1C***).

### H_2_-rich saline treatment decreases production of proinflammatory cytokines

**Fig. 2:**
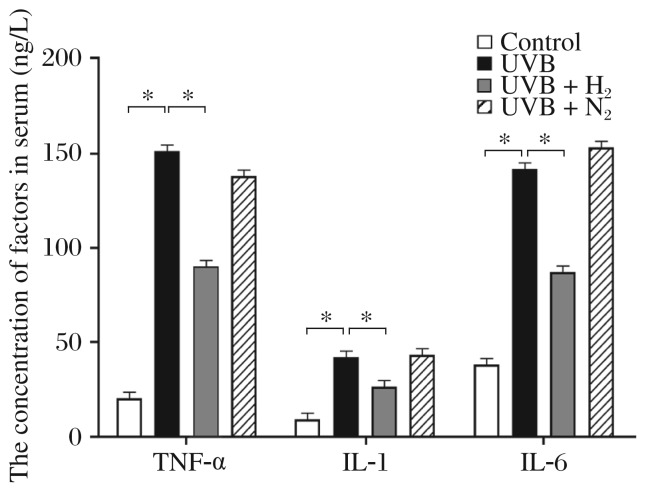
Serum concentrations of TNF-α, IL-1β and IL-6 in controls, UVB irradiated rats, and UVB irradiated rats treated with H_2_-rich saline or nitrogen-rich saline. *P < 0.05.

As shown in ***Fig. 2*** and ***Table 1***, tumor necrosis factor alpha (TNF-α), interleukin (IL)-1β and IL-6 were also detected in serum. These proteins were significantly increased after UVB irradiation compared with the control group. Administration of H_2_-rich saline intraperitoneally lowered the UVB irradiation-induced elevation of TNF-α, IL-1β and IL-6 serum concentrations. No significant reduction of these markers was observed in nitrogen-rich saline.

### H_2_-rich saline reduces oxidative stress

**Fig. 3:**
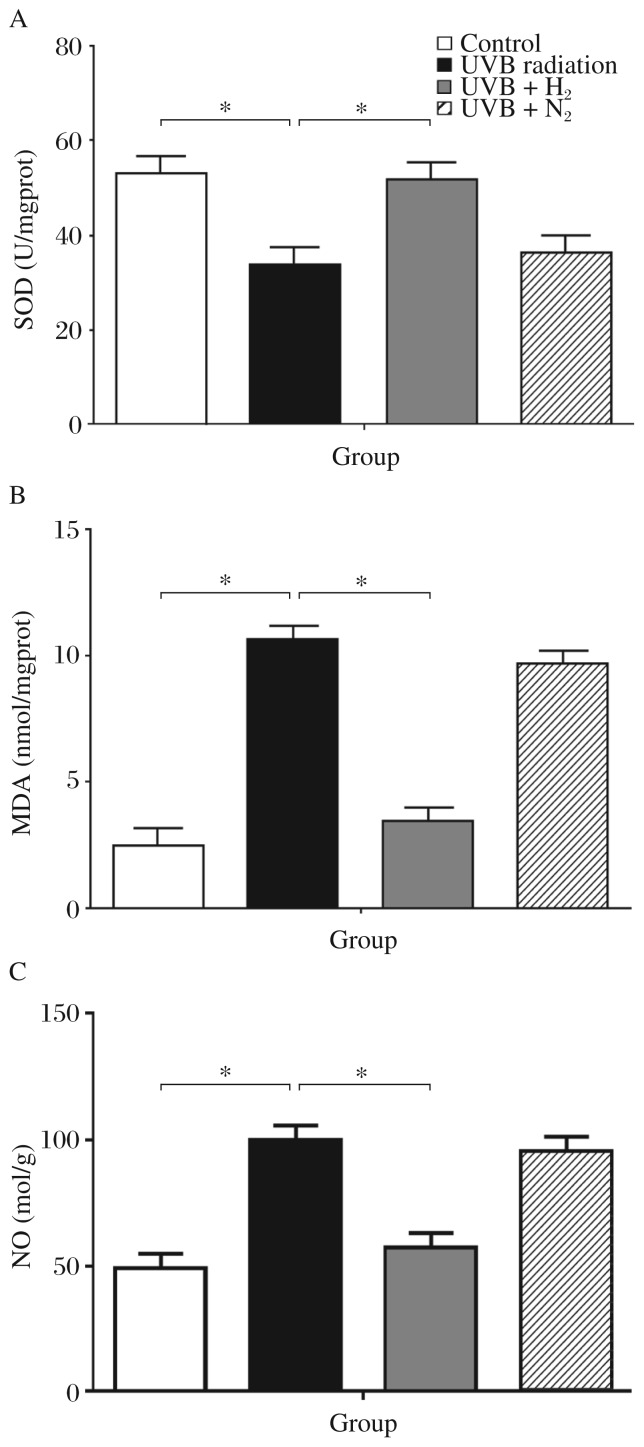
Skin superoxide dismutase (SOD), mucosal malondialdehyde (MDA) and nitric oxide (NO) level in controls, UVB irradiated rats, and UVB irradiated rats treated with hydrogen-rich saline or nitrogen-rich saline. Data are expressed as mean±SD for at least triplicate independent experiments (*n* = 8 per group). *P < 0.05.

SOD, MDA and NO activities as indicators of oxidative stress are shown in ***Fig. 3***. The group without treatment showed the highest levels of SOD and the lowest MDA and NO expression. Rats subjected to UVB irradiation alone showed a significant decrease of SOD activities compared with the control group (UVB *vs* Control: 33.48±4.28 *vs* 52.43±6.56, *P* < 0.05). The group that received H_2_-rich saline treatment showed a significant increased in SOD activities compared to the untreated irradiated group (51.81±5.67 *vs* 33.48±4.28, *P* < 0.05). The nitrogen-rich saline group showed no noticeable decrease in SOD activity (***Fig. 3A***). UVB radiation injury caused a significant increase of MDA in the untreated group more than the control group (10.69±0.72 *vs* 2.5±0.6, *P* < 0.01).

Administration of H_2_-rich saline resulted in a marked reduction of MDA levels compared to the untreated group (3.39±0.71 *vs* 10.69±0.72, *P* < 0.01). Nitrogen-rich saline did not reduce the MDA level significantly (***Fig. 3B***). Similar results were obtained for NO. The group treated with H_2_-rich saline showed the most significant increase of NO levels compared to the untreated group (58.48±6.38 *vs* 100.2±9.56, *P* < 0.01). Nitrogen-rich saline did not reduce NO levels significantly (***Fig. 3C***).

## DISCUSSION

This is the first study on the use of H_2_-rich saline to alleviate acute UVB radiation injury in an animal model. We successfully established an acute UVB injury model and found that H_2_-rich saline had therapeutic antioxidant activity in this model. We principally investigated the effect of H_2_-rich saline on oxidative stress and inflammation.

After absorbing UV radiation, molecules may become altered (damaged) and/or affect (damage) other molecules, e.g. by producing ROS. Thus, UV radiation, especially in the high-energy, short wavelength range, forms a direct threat to the stability of organic molecules that are essential to life on earth. Both UVB and UVA radiation can cause ROS generation and ROS-mediated damage[19]. This damage appears to arise indirectly through UV absorption by other “endogeneous photosensitizers”, which then form radicals that can react with DNA. ROSs appear to play a prominent role in these radical reactions[20].

By histochemical staining, we found that H_2_-rich saline decreased neutrophil infiltration, a typical indicator of an acute inflammatory reaction. In skin tissue subjected to UVB irradiation, activated inflammatory cells induce tissue injury mainly through the production and release of ROSs[21]–[23]. After UVB irradiation of skin tissue, we found massive neutrophil infiltration, principally in the hypodermis. However, with intraperitoneal injection of H_2_-rich saline, neutrophil infiltration was not as apparent. Nitrogen-rich saline produced no significant reduction of inflammation. The severity of inflammation were was graded semi-quantitatively by light microscopy, and we can conclude from the data that H_2_-rich saline can reduce the infiltration of neutrophils. These results are consistent with those of a published study on rat intestine[24]. We did not find any statistical difference in skin thickness between these groups, suggesting that the thickening of skin tissue caused by edema was not achieved by a single dose of UVB irradiation[25],[26].

The role of H_2_-rich saline in ameliorating UVB-induced inflammatory response is supported by the finding of higher levels of proinflammatory cytokines TNF-α, IL-1β and IL-6. Elevation of these cytokines can cause inflammatory cell infiltration into damaged tissues[27]. UVB irradiation of rat skin caused a significant elevation of TNF-α, IL-1β and IL-6 in serum, which was reduced by H_2_-rich saline administration. This suggests that H_2_-rich saline treatment may protect against acute UVB damage by inhibiting skin inflammatory response.

Oxidative damage plays an important role in UVB radiation injury by damaging DNA, inactivating enzymes, causing damage to the structure and function of cell membranes, and killing certain cells, ultimately leading to cell aging and degradation[28]. Direct UVB radiation of fibroblasts can produce oxygen free radicals, which attack polyunsaturated fatty acids on the cell membrane, leading to lipid peroxidation and the formation of lipid peroxidation products, thereby causing cell damage[29]. SOD activity can reflect the body's ability to eliminate oxygen free radicals. MDA is produced during the attack of free radicals on membrane lipoproteins and polyunsaturated fatty acids, and MDA levels can reflect the local extent of lipid peroxidation and cell injury. MDA levels are often comparable to those of SOD, and both were associated with oxidative skin tissue damage in our animal model. NO, which mediates the pathological function of such factors as endotoxin, TNF-α, IL-1β and IL-6, was also increased in our study.

We found that H_2_-rich saline might reduce oxidative stress by altering the expression of these factors, which might be another protective mechanism[30]. In several recent studies, H_2_ inhalation has been reported to protect against ischemia-reperfusion injury by reducing oxidative stress in animal models[10],[11]. Nagata showed that, in mice subjected to prolonged physical restraint, drinking hydrogen-rich water can reduce oxidative stress in the brain and prevent stress-induced declines in learning and memory[31]. Furthermore, lipid and glucose metabolism in patients with type 2 diabetes or impaired glucose tolerance can be reduced by drinking hydrogen-rich water[32].

In this study, we demonstrated that molecular hydrogen might reduce inflammation and oxidative stress in the skin after UVB irradiation. Injection of H_2_-rich saline into the skin following UVB irradiation is safe and easy to administer in clinical use. We tested the effects of nitrogen-rich saline on our UVB irradiation model. Nitrogen-rich saline, which is also a deoxygenated solution, was prepared in the same way as H_2_-rich saline. Nitrogen-rich saline treatment did not reduce oxidative stress or the inflammatory response following UVB irradiation, suggesting that the observed protection conferred by H_2_-rich saline was mediated via an antioxidant action.

In conclusion, intraperitoneal injection of H_2_-rich saline protected rat skin tissue against UVB radiation injury, possibly by reduction of inflammation and oxidative stress. Other methods of administration, such as local application, should be considered, but the most suitable carrier for H_2_-rich saline is still under research. It also remains to be determined whether H_2_-rich saline can delay skin aging caused by UV radiation through reducing ROS and the inflammatory reaction[26],[33],[34]. The exact mechanism and signaling pathway involved in the protective role of H_2_ against UVB radiation injury will be addressed in future studies.
